# Supplementary material for: Protein Hydration in a Bioprotecting Mixture
Source: Life (Basel). 2021 Sep 22;11(10):995. doi: 10.3390/life11100995 (PMC8537178; doi:10.3390/life11100995)
Supplement: Supplementary file 1 [file life-11-00995-s001.zip › life-1343651-supplementarynew.pdf]

life

MDPI

Supplementary

Protein Hydration in a Bioprotecting Mixture

Silvia Corezzi[1], Brenda Bracco[2], Paola Sassi[2], Marco Paolantoni[2,]* and Lucia Comez[3,]*

[1] Dipartimento di Fisica e Geologia, Università degli Studi di Perugia
[2] Dipartimento di Chimica, Biologia e Biotecnologie, Università degli Studi di Perugia
[3] CNR-IOM, c/o Dipartimento di Fisica e Geologia, Università degli Studi di Perugia
* Correspondence: comez@iom.cnr.it, marco.paolantoni@unipg.it

S1. Normalization procedure of the EDLS spectra

The normalization procedure of the EDLS spectra of ternary (WTL) (3% in weight of lysozyme and 40% trehalose) and binary WT (40% in weight of trehalose) & WL (4% in weight of lysozyme) solutions was performed as shown in Figure S1.

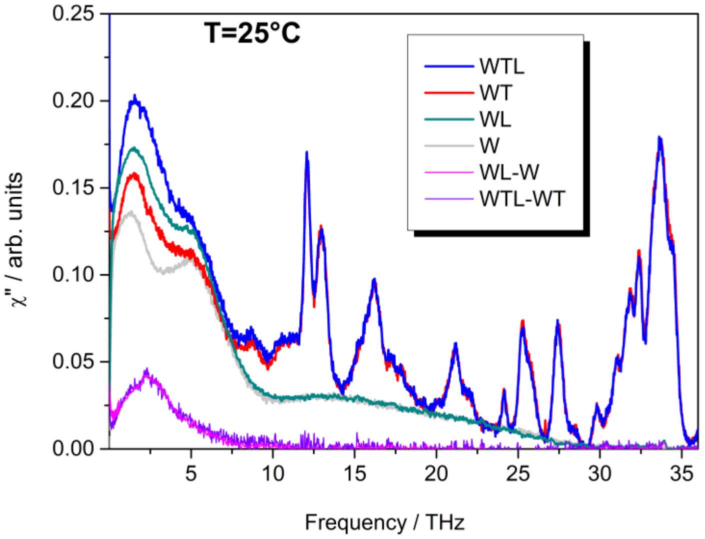

Figure S1. EDLS spectra of WTL and WT mixtures represented on a Lin-Lin scale to better visualize the normalization of WTL mixtures to the high-frequency peaks (> 10 THz) corresponding to the Raman active modes of trehalose. It should be noted that these high-frequency peaks lie on the librational band of the spectrum of pure water recorded at the same temperature, which is also common to the WL solution (50 mg/ml). The difference spectra (WL-W and WTL-WT) generated using such a normalization are also shown in the figure, showing perfect superimposition of the vibrational modes of lysozyme, and providing independent proof of the goodness of the scaling procedure carried out.

S2. EDLS spectral components of the water lysozyme solution at 50 mg/ml and 25°C

A two-step global fitting procedure was applied in order to rationalize the complex EDLS spectral distributions of the lysozyme aqueous (WL) solution at 50 mg/ml (almost the same protein content in the ternary mixture), thus providing a consistent modeling of the whole spectrum as shown in Fig. S2. The entire procedure is detailed in Ref. [2].

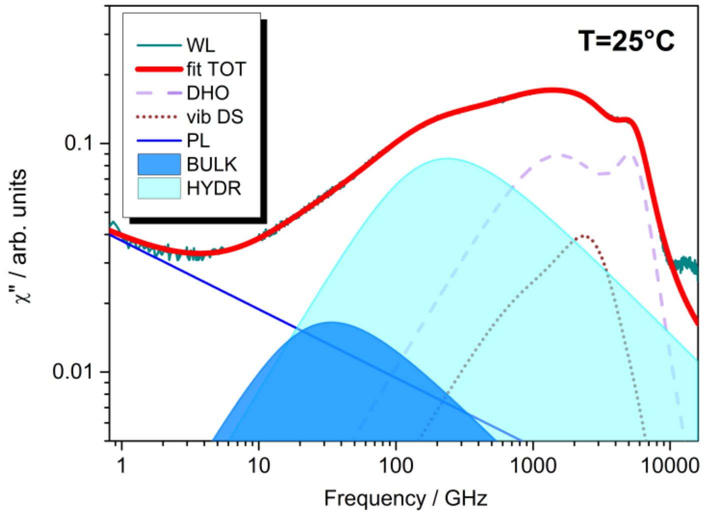

Figure S2. EDLS susceptibility spectra for WL solution (lysozyme/water 50 mg/ml) at 25°C. Experimental and global best fit curves with individual components are showed: PL, the power law ($\nu^{-0.3}$) describing the tail of lysozyme relaxation [1-4]; DHO, given by the sum of inter-molecular water bending and stretching modes, vib DS, the vibrational contribution (arising from lysozyme) reproduced with three Brownian oscillators as in Ref.[2], and finally BULK and HYDR, hydration and bulk water relaxations, respectively, modeled by two CD functional forms, with the shape parameter fixed to 0.6 as for the pure water [2].

S3. EDLS difference spectra

The method to reproduce the EDLS susceptibility profile of the difference spectra, $\chi_{DS}"(\nu)$, over the entire frequency range from ~1 GHz to ~10 THz (Fig. S3 a), thus estimating the residual water contribution in WTL mixtures, was described in detail in Ref. [1]. It was found that the following sum of different components is able to realistically model the spectra:

$$\chi_{DS}"(\nu) = A\nu^{-0.3} + \Delta_{res}\frac{2\pi\nu\tau_{res}}{[1+(2\pi\nu\tau_{res})^2]} + \sum_{i=1}^{3} BO_i(\nu) \qquad (1)$$

where A is a constant, Δ_{res} and τ_{res} are the amplitude and the characteristic time of the Debye relaxation, and $BO_i(\nu)$ with i=1…3 represent BO lineshapes. As an example, the best-fit curves obtained for the WTL solution at 25°C are shown in Fig. S3 b. In the same panel, the results for WL at the same temperature are also displayed. It emerges that the relaxation process of the residual water in the ternary solution is actually slowed down over twice compared to the binary protein solution, demonstrating the existence of a component of *ultraslow* water when trehalose is present in the system. It is important to note that, although the functional form that best represents the water relaxation behavior is a stretched Cole-Davidson (CD) function, the data of WTL solutions do not allow us to discriminate between the two forms. Therefore, we have chosen to analyze the experimental DS by using the minimum number of free-fit parameters, after verifying that at ambient temperature the use of Debye and CD equations provides comparable values for the average relaxation time of the process.

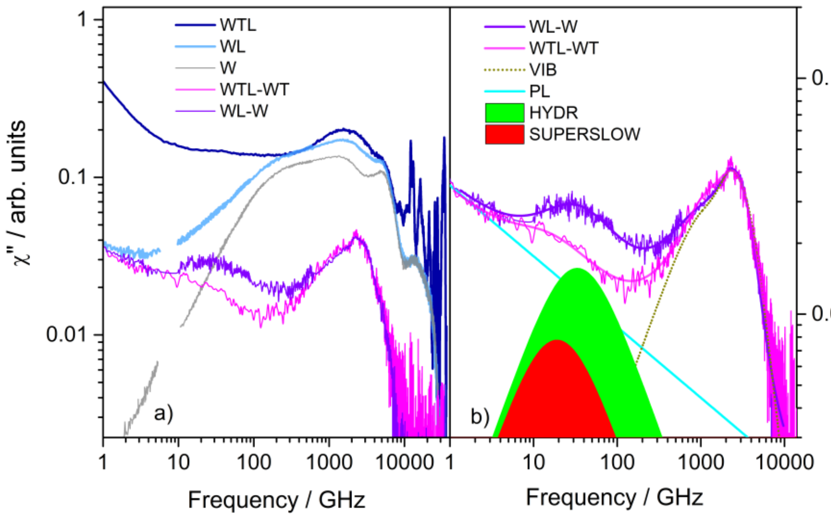

Figure S3. (**a**) EDLS spectra of WTL and WL mixtures collected at ambient temperature, represented on a Log-Log scale. The spectrum of pure water (W) and the difference spectra (DS) of WTL-WT and WL-W solutions (T=25°C) are also reported in the same picture. (**b**) DS spectra WTL-WT and WL-W together with the best-fitting total curves, obtained according to eq. 1. The power-law con-tribution (PL), Debye water relaxations, and vibrational (sum of 3 BO) component (VIBR) are sepa-rately indicated.

References

[1] Corezzi, S.; Paolantoni, M.; Sassi, P.; Morresi, A.; Fioretto, D.; Comez, L. Trehalose-Induced Slowdown of Lysozyme Hydration Dynamics Probed by EDLS Spectroscopy. *J. Chem. Phys.* **2019**, *151*, 015101.

[2] Perticaroli, S.; Comez, L.; Paolantoni, M.; Sassi, P.; Lupi, L.; Fioretto, D.; Paciaroni, A.; Morresi, A. Broadband Depolarized Light Scattering Study of Diluted Protein Aqueous Solutions. *The Journal of Physical Chemistry B* **2010**, *114*, 8262–8269.

[3] Khodadadi, S.; Pawlus, S.; Sokolov, A.P. Influence of Hydration on Protein Dynamics: Combining Dielectric and Neutron Scattering Spectroscopy Data. *The Journal of Physical Chemistry B* **2008**, *112*, 14273–14280.

[4] Roh, J.H.; Curtis, J.E.; Azzam, S.; Novikov, V.N.; Peral, I.; Chowdhuri, Z.; Gregory, R.B.; Sokolov, A.P. Influence of Hydration on the Dynamics of Lysozyme. *Biophysical Journal* **2006**, *91*, 2573–2588.
